# Cardiovascular magnetic resonance stress perfusion imaging predicts 1 year outcomes following equivocal stress testing

**DOI:** 10.1186/1532-429X-14-S1-P9

**Published:** 2012-02-01

**Authors:** Timothy C Wong, Diego Moguillansky, Kathryn Berlacher, Erik Schelbert

**Affiliations:** 1 Heart and Vascular Institute, University of Pittsburgh School of Medicine, Pittsburgh, PA, USA

## Summary

We tested the hypothesis that cardiovascular magnetic resonance (CMR) stress perfusion can predict 1 year outcomes in individuals with equivocal or uncertain prior stress testing.

## Background

Individuals with equivocal nuclear stress test results are at higher risk for cardiac events compared to those with normal studies. CMR stress perfusion imaging identifies individuals with coronary artery disease with high sensitivity and specificity, with excellent spatial resolution, and without ionizing radiation. The utility of CMR stress testing is uncertain in individuals with prior equivocal stress results.

## Methods

We selected all participants in a CMR registry who were clinically referred for pharmacologic CMR perfusion testing due to recent nuclear stress perfusion study (6 months if outpatient, same admission if inpatient) from 2009-2010 with equivocal or uncertain results. Studies were defined as equivocal if the results were qualified by the mention of significant artifact or other technical difficulty. Studies were defined as uncertain if the referring physician documented suspicion of false positive or false negative results in the medical record. The presence of ischemia reported by each modality, as well as any description of uncertainty, was determined by a clinical nurse reviewer and 2 cardiologists (all blinded to the downstream clinical course of each subject). Any disagreement in assessment was arbitrated by majority vote. The clinical course of each subject was followed through chart review. Adverse outcome was defined as hospitalization for suspected or definite myocardial infarction.

## Results

19 individuals met inclusion criteria: mean age 58, female = 7, indications for initial stress testing were either symptoms suspicious for coronary artery disease or preoperative evaluation. CMR reclassified 13 (68%) individuals: a) among the 11 with likely ischemia on nuclear stress testing, but none by CMR, no adverse events were noted during an average follow up of 12 months; b) among the 2 without likely ischemia on nuclear stress testing but significant ischemia on CMR stress test, one received percutaneous coronary intervention and the other opted for medical therapy. Among individuals with concordant nuclear and CMR findings, all 3 with significant ischemia underwent revascularization following diagnostic coronary angiography, and no adverse events were noted in the 3 without ischemia.

## Conclusions

Following equivocal or uncertain nuclear stress testing, CMR stress perfusion reclassified a high proportion of patients with favorable 1 year outcomes. Further studies in larger populations should be performed.

## Funding

Dr. Wong is supported by a grant K12 HS19461-01 from the Agency for Healthcare Research and Quality. Dr. Schelbert is supported by a Pittsburgh Foundation grant and an American Heart Association Scientist Development grant (09SDG2180083) including a T. Franklin Williams Scholarship Award; funding provided by: Atlantic Philanthropies, Inc, the John A. Hartford Foundation, the Association of Specialty Professors, and the American Heart Association.

